# Electrical Pulse Stimulation Partially Restores Insulin Signaling in Palmitate‐Induced Insulin‐Resistant Skeletal Muscle Cells via Mitochondrial Remodeling and Contractile Activity‐Dependent Secretome

**DOI:** 10.1002/jcp.70240

**Published:** 2026-10-06

**Authors:** Falcone Alessandro, Yilmaz Sevgican, Hendlinger Mona, Schrauwen Patrick, Lipaeva Polina, Piribauer Marlene, Heilmann Geronimo, Trenkamp Sandra, Roden Michael, Mastrototaro Lucia

**Affiliations:** ^1^ Institute for Clinical Diabetology, German Diabetes Center, Leibniz Center for Diabetes Research Heinrich‐Heine‐University Düsseldorf Germany; ^2^ German Center for Diabetes Research (DZD e.V.) Partner Düsseldorf Neuherberg Germany; ^3^ Department of Endocrinology and Diabetology, Medical Faculty and University Hospital Düsseldorf Heinrich‐Heine‐University Düsseldorf Düsseldorf Germany

**Keywords:** exercise, insulin resistance, mitochondrial dynamics, myokines, skeletal muscle cells

## Abstract

Lipid oversupply induces insulin resistance in skeletal muscle (SkM), partly by altering mitochondrial function and dynamics. Although electrical pulse stimulation (EPS) improves insulin signaling in myotubes, the mechanisms contributing to this effect under lipotoxic conditions remain unclear. Thus, we investigated whether EPS restores lipid‐induced insulin resistance in association with changes in mitochondrial respiration and dynamics, lipotoxic intermediates and evaluated the contribution of contraction‐induced secreted factors, including small extracellular vesicles (sEV). Murine C2C12 myotubes were treated with palmitate (PA, 0.3 mmol/l) and subjected to EPS (1 Hz, 11.5 V) for 24 h. Insulin signaling, mitochondrial dynamics, respiratory capacity and lipotoxic intermediates were assessed by Western blotting, high‐resolution respirometry and liquid chromatography‐tandem mass spectrometry in control (CON) and PA‐treated cells before and after EPS. Additionally, CON and PA‐treated cells were incubated for 24 h with conditioned media and sEV from unstimulated or EPS‐stimulated CON and PA‐treated cells to evaluate the effect of the myocellular secretome on AKT signaling. PA reduced insulin‐stimulated AKT(Ser473) and (Thr308) phosphorylation, which was partially rescued by EPS. In PA cells, EPS decreased the fusion proteins mitofusin 1 and 2 (MFN1, MFN2) and increased the fission marker dynamin‐related protein 1 (DRP1). However, mitochondrial respiration and lipid accumulation were unaffected by EPS. Furthermore, conditioned media and sEV from EPS‐stimulated cells enhanced AKT phosphorylation in PA‐treated cells. These findings demonstrate that attenuation of lipid‐induced insulin resistance is independent of mitochondrial respiratory capacity and lipid accumulation, but it is associated with alterations in proteins involved in mitochondrial dynamics and is likely mediated by exercise‐derived sEV.

## 1 Introduction

Skeletal muscle (SkM) insulin resistance is a hallmark of type 2 diabetes mellitus (T2DM) and strongly associates with ectopic lipid accumulation and reduced mitochondrial function in sedentary individuals (Roden and Shulman 2019). Indeed, human studies suggest that altered mitochondrial oxidative capacity and dynamics, as well as activated stress pathways such as endoplasmic reticulum (ER) stress may also contribute to lipid‐induced insulin resistance in SkM (Han and Kaufman 2016; Fiorenza et al. 2024). In turn, lipid accumulation in SkM reduces features of mitochondrial function (Hoeks et al. 2010; Chavez et al. 2010), which could be due to induction of dynamin‐related protein 1 (DRP1)‐mediated fragmentation, suggesting a mechanistic link between lipid accumulation, mitochondrial remodeling and impaired insulin signaling (Axelrod et al. 2021). People with T2DM exhibit also mitochondrial fragmentation and reduced levels of the fusion proteins, mitofusin 2 (MFN2) and optic atrophy 1 (OPA1) (Joseph et al. 2012; Gundersen et al. 2020), thereby contributing to impaired mitochondrial functionality and insulin resistance and supporting the relevance of mitochondrial remodelling (Axelrod et al. 2021; Gundersen et al. 2020; Dantas et al. 2025; Houzelle et al. 2021).

Exercising is an effective strategy to improve SkM insulin sensitivity (Röhling et al. 2016; Sigal et al. 2006). Exercise training promotes mitochondrial biogenesis, oxidative capacity, and leads to a more fused mitochondrial tubular network, increased mitochondrial respiration and insulin sensitivity in insulin‐resistant individuals with and without T2DM (Ruegsegger et al. 2023; Little et al. 2011; Mastrototaro et al. 2024). Importantly, acute exercise can also reverse lipid‐induced insulin resistance in human SkM (Smiles et al. 2019) and increases the interaction between lipid droplets and mitochondria, thereby favoring fatty acid oxidation (de Almeida et al. 2023). Conversely, physical inactivity reduces mitochondrial oxidative capacity, leading to increased intramyocellular lipid (IMCL) accumulation and insulin resistance (Bilet et al. 2020), thus highlighting the importance of contraction‐induced mitochondrial adaptation. How exercise improves lipid‐induced insulin resistance and mitochondrial respiration and dynamics is not fully understood, but may also involve the secretion of contraction‐dependent factors, which can affect insulin sensitivity, inflammation and systemic metabolism (Mastrototaro and Roden 2024).

Electrical pulse stimulation (EPS) has been established as an in vitro model to mimic contraction‐induced signaling in cultured muscle cells (Nikolić et al. 2017). Previous studies have shown that EPS can improve insulin signaling in insulin‐resistant myotubes, including palmitate (PA)‐treated C2C12 cells and primary human SkM cells from lean and severely obese donors (Nieuwoudt et al. 2017; Kugler et al. 2021; Park et al. 2019).

However, the mechanisms underlying these beneficial effects under lipotoxic conditions remain incompletely understood. In particular, it remains unclear to what extent the restoration of insulin signaling is mediated by intracellular adaptations, including changes in lipid accumulation, mitochondrial function, mitochondrial dynamics and ER stress or by contraction‐induced muscle‐derived soluble factors, including small extracellular vesicles (sEV). Indeed, muscle‐derived sEV have been shown to mediate pro‐metabolic effects in recipient cells, resembling the effects of exercise (Obi et al. 2025). Thus, the aim of the present study was to determine whether EPS restores AKT signaling in PA‐induced insulin‐resistant C2C12 myotubes through intracellular adaptations and/or via contraction‐induced muscle‐derived sEV. We hypothesized that EPS improves insulin signaling under lipotoxic conditions through changes in proteins involved in mitochondrial dynamics and the release of bioactive factors.

## 2 Materials and Methods

### 2.1 Cell Culture and Differentiation

C2C12 myoblasts were cultured in Dulbecco's Modified Eagle Medium (DMEM; Gibco, ThermoFisher Scientific, Waltham, USA; Cat. No.: 11955), containing 4.5 g/l d‐glucose and l‐glutamine and supplemented with 10% fetal bovine serum (FBS; Merck, Darmstadt, Germany; Cat. No.: ES‐009‐C) and 1% penicillin/streptomycin (P/S; 10,000 U/mL; Gibco, ThermoFisher Scientific; Cat. No.: 15140122), at 37°C in a humidified incubator, as previously described (Lambernd et al. 2012). Growth medium (GM) was changed every 2 days.

For differentiation, cells were seeded at a density of 2 × 10^5^ cells per well into 6‐well plates (Figure S1A). For each independent experiment, cells were derived from a single cell batch and plated in parallel into four plates, one for each experimental conditions (CON, PA, EPS, PA + EPS). Upon reaching 90–100% confluency (day 0), GM was replaced with differentiation medium (DM; DMEM supplemented with 2% Horse Serum (Gibco, ThermoFisher Scientific; Cat. No.: 16050130) and 1% P/S) to induce myogenic differentiation (Lambernd et al. 2012). DM was changed every other day. Protein content of myosin heavy chain 3 (MYH3) was assessed with immunoblot as a marker of differentiation for mature myotubes (Lambernd et al. 2012). On day 5, cells were incubated overnight in serum‐free starvation medium (SM; DMEM supplemented with 1% P/S) to minimize confounding effects of serum‐derived factors. On day 6, following overnight serum starvation, cells were treated under serum‐free conditions with BSA (CON), palmitate (PA), electrical pulse stimulation (EPS), or a combination of both (PA + EPS) for 24 h and harvested on day 7. Alternatively, at day 6 differentiated CON and PA myotubes were incubated with conditioned media (CM) from CON (CM‐CON) or EPS‐stimulated (CM‐EPS) C2C12 myotubes. Viability of the cells was assessed by morphological inspection and by measuring the lactate dehydrogenase (LDH) release into the culture media (CyQUANT™ LDH Cytotoxicity Assay Kit, ThermoFisher Scientific; C20300), according to the manufacturer instructions.

### 2.2 Palmitate Treatment

A PA (Sigma, P9767) stock solution (20 mmol/l) was prepared in 0.1 M NaOH (Sigma, S8045) at 70°C and conjugated to bovine serum albumin (BSA, Carl Roth, 0052.3) to generate a 5 mmol/l PA working solution (Listenberger et al. 2001). Control solution, used for CON and EPS group, contained equivalent concentrations of NaOH and BSA. PA‐BSA and BSA control solutions were diluted in prewarmed SM to a final PA concentration of 0.3 mmol/l immediately before use. Cells were incubated with PA‐BSA (PA) or BSA (CON) for 24 h.

### 2.3 Electrical Pulse Stimulation

EPS was applied to fully differentiated myotubes in a 6‐well dish using a C‐Dish electrode system combined with a pulse generator (C‐Pace 100; IonOptix, Milton, MA, USA), as previously described (Lambernd et al. 2012). Bipolar electrical pulses were applied at a frequency of 1 Hz with a pulse duration of 2 ms at continuous chronic low‐frequency (1 Hz) and moderate voltage of 11.5 V for 24 h, resulting in rhythmic contractions of the myotubes (Video S1) (Lambernd et al. 2012). CON cells were exposed to electrodes which were not connected to the power supply. After EPS, the cell culture supernatant was collected for analysis of interleukin‐6 (IL‐6) content with the BioVendor Mouse Interleukin‐6 ELISA Kit (Lambernd et al. 2012). Furthermore, the supernatant of CON and EPS cells as well as PA and PA + EPS cells was collected and centrifuged at 2000 x g for 5 min at 4°C to remove debris and apoptotic cells and used as CM to assess the effect of secreted factors on insulin signaling of CON and PA cells.

### 2.4 Small Extracellular Vesicles (sEV) Isolation From Myotubes

sEV were isolated from differentiated myotubes of the 4 experimental groups (CON, EPS, PA, PA + EPS). The medium was collected, centrifuged at 300 × *g* for 5 min, and sEV were isolated by ultrafiltration coupled to successive differential ultracentrifugation, as previously described (Hartwig et al. 2019). Briefly, the supernatant was first cleared at 2000 × *g* for 20 min followed by centrifugation at 10,000 × *g* for 30 min. Supernatant was then filtrated through a 0.2 μm filter. After filtration, the culture medium was centrifuged at 100.000 ×*g* for 70 min. The transparent pellet containing sEV was resuspended in PBS and used for treatment of target cells.

### 2.5 Insulin Stimulation

For analysis of insulin signaling, cells were incubated with fresh serum‐free medium containing insulin (Insulin from bovine pancreas powder, BioReagent, suitable for cell culture, Merck; Cat. No.: I6634) at a final concentration of 100 nM for 15 min following the 24 h PA and/or EPS treatment (Lambernd et al. 2012).

### 2.6 Protein Extraction and Immunoblotting

The content of proteins of interest was assessed by Western blotting (WB) as previously described (Mastrototaro et al. 2024). Cells were lysed with lysis buffer supplemented with protease (cOmplete Tablets, EASYpack, Roche Diagnostics, Basel, Switzerland) and phosphatase (PhosSTOP, EASYpack, Roche Diagnostics) inhibitors (Mastrototaro et al. 2024. Lysates were cleared by centrifugation (14,000 rpm, 15 min, 4°C), and protein concentration was determined using a bicinchoninic acid (BCA) assay. Equal amounts of protein were diluted with reducing Laemmli sample buffer containing 2‐mercaptoethanol (1610747, Bio‐Rad, CA, USA), boiled 5 min at 95°C and loaded onto a SDS‐polyacrylamide gradient gel (4%–20% Mini‐PROTEAN TGX Precast Protein Gels, Bio‐Rad, CA, USA). Following electrophoresis, the gels were UV‐activated and proteins were transferred to a polyvinylidene difluoride membrane using the Trans‐Blot Turbo Transfer System (Bio‐Rad, CA, USA). Membranes were blocked for 2 h at room temperature with 5% BSA or milk in tris‐buffered saline with Tween (TBST) and incubated overnight at 4°C with primary antibodies (Supplementary Table 1). Next day, membranes were washed with TBST buffer and incubated with horseradish peroxidase–conjugated secondary antibodies (Supplementary Table 1). Finally, the membranes were coated with Immobilon Western Chemiluminescent HRP substrate (Millipore, Danvers, MA, USA), and the proteins were visualized using a Bio‐Rad ChemiDoc MP Imaging System in combination with the software ImageLab 6.0.1 (Bio‐Rad, CA, USA) for densitometric analysis. Data are expressed in arbitrary units and normalized to total protein content (Supplemental Western Blot file).

### 2.7 High‐Resolution Respirometry (HRR)

Mitochondrial respiration was measured in duplicates in MiR05 at 37°C, 750 rpm stirrer speed and a chamber volume of 2.0 ml using the high‐resolution Oxygraph‐2k (Oroboros Instruments, Innsbruck, Austria) and a substrate‐uncoupler‐inhibitor (SUIT) protocol, as previously described (Pafili et al. 2022). Cellular membranes were permeabilized by digitonin (4 μmol/l) in the chamber. Leak respiration (L) with electron input through electron transferring flavoprotein (ETF) ([ETF]_L_) was determined after addition of malate (0.1 mmol/l) and octanoyl‐carnitine (0.2 mmol/l). Adenosine diphosphate (ADP) (2.5 mmol/l) was added to determine oxidative phosphorylation (OXPHOS) capacity (P) with the same electron input ([ETF]_P_). Cytochrome c (10 μmol/l) was added to test the integrity of the outer mitochondrial membrane and chambers presenting an increase greater than 15% were excluded. Subsequent addition of glutamate (10 mmol/l) and titration of succinate was used to assess P with convergent electron input through ETF and complex (C)I ([ETF + CI]_P_) or ETF, CI and CII combined ([ETF + CI + II]_P_). L and electron transport system capacity (E) with the same convergent electron input ([ETF + CI + II]_L_ and [ETF + CI + II]_E_) were measured after addition of oligomycin (5 μM) and by titration with carbonyl cyanide p‐[trifluoromethoxyl]‐phenyl‐hydrazone (FCCP), respectively. Finally, non‐mitochondrial‐driven respiration was measured after addition of antimycin A (5 μmol/l). O_2_ flux rates were corrected for number of cells (400.000/chamber) and were expressed as pmol * n cells^−1^ * s^−1^.

#### 2.7.1 Liquid Chromatography‐Tandem Mass Spectrometry (LC‐MS/MS)

CER and DAG were quantified by liquid chromatography‐tandem mass spectrometry as previously described, with minor modifications (Preuss et al. 2019). Cells were weighted and homogenized, internal standards were added, and lipids were extracted using the Folch method (Folch et al. 1957). Samples were separated by reverse‐phase ultra‐high‐performance liquid chromatography and analyzed using a triple quadrupole mass spectrometer operated in positive ion mode. Data acquisition and processing were performed using MassHunter software. CER and DAG concentrations were normalized to cell weight in gram.

### 2.8 Statistical Analysis

Graphs were generated using GraphPad Prism® software (GraphPad Software, 9.5.1, San Diego, CA, USA). Right‐skewed data were log‐transformed prior to analysis. Normality was assessed using the Shapiro–Wilk test. For normally distributed data, one‐way repeated‐measures ANOVA or one‐way ANOVA for experiments involving CM and sEV were performed, followed by pairwise paired t‐tests. For data that did not meet normality assumptions, non‐parametric analyses were conducted using the Friedman test followed by paired Wilcoxon signed‐rank tests or the Kruskal–Wallis test followed by Dunn test. Multiple‐comparison adjustment was performed using the Bonferroni correction. Statistical significance was defined as an adjusted *p*‐value < 0.05.

## 3 Results

### 3.1 Establishment and Validation of the C2C12 Insulin Resistance Model

To determine the myogenic differentiation, protein levels of MYH3 were measured in myoblasts and mature myotubes. After 6 days of differentiation, C2C12 cells displayed higher levels of MYH3 protein compared to myoblasts regardless of the initial seeding density (Figure S1B). However, starting with a seeding density of 200,000 myoblasts per well resulted in the highest MYH3 content and this density was therefore used for all subsequent experiments. Additionally, differentiation of mononucleated myoblasts into multinucleated myotubes was confirmed using bright‐field imaging (Figure S1C).

PA cytotoxicity was dose‐dependent, as evidenced by 18% and 42% elevation in LDH release at 0.3 and 0.4 mmol/l PA, respectively, compared with CON. In contrast, LDH release in cells treated with 0.2 mM PA did not differ from CON (Figure 1A). Next, we examined whether PA affected insulin signaling by measuring AKT(Ser473) phosphorylation. We found that PA 0.4 and 0.3 mmol/l induced a 2.3‐ and 1.5‐fold reduction in AKT(Ser473) phosphorylation, respectively (Figure 1B). Since 0.3 mmol/l PA effectively induced insulin resistance with minimal cytotoxicity, all subsequent experiments were performed using this concentration of PA.

**Figure 1:**
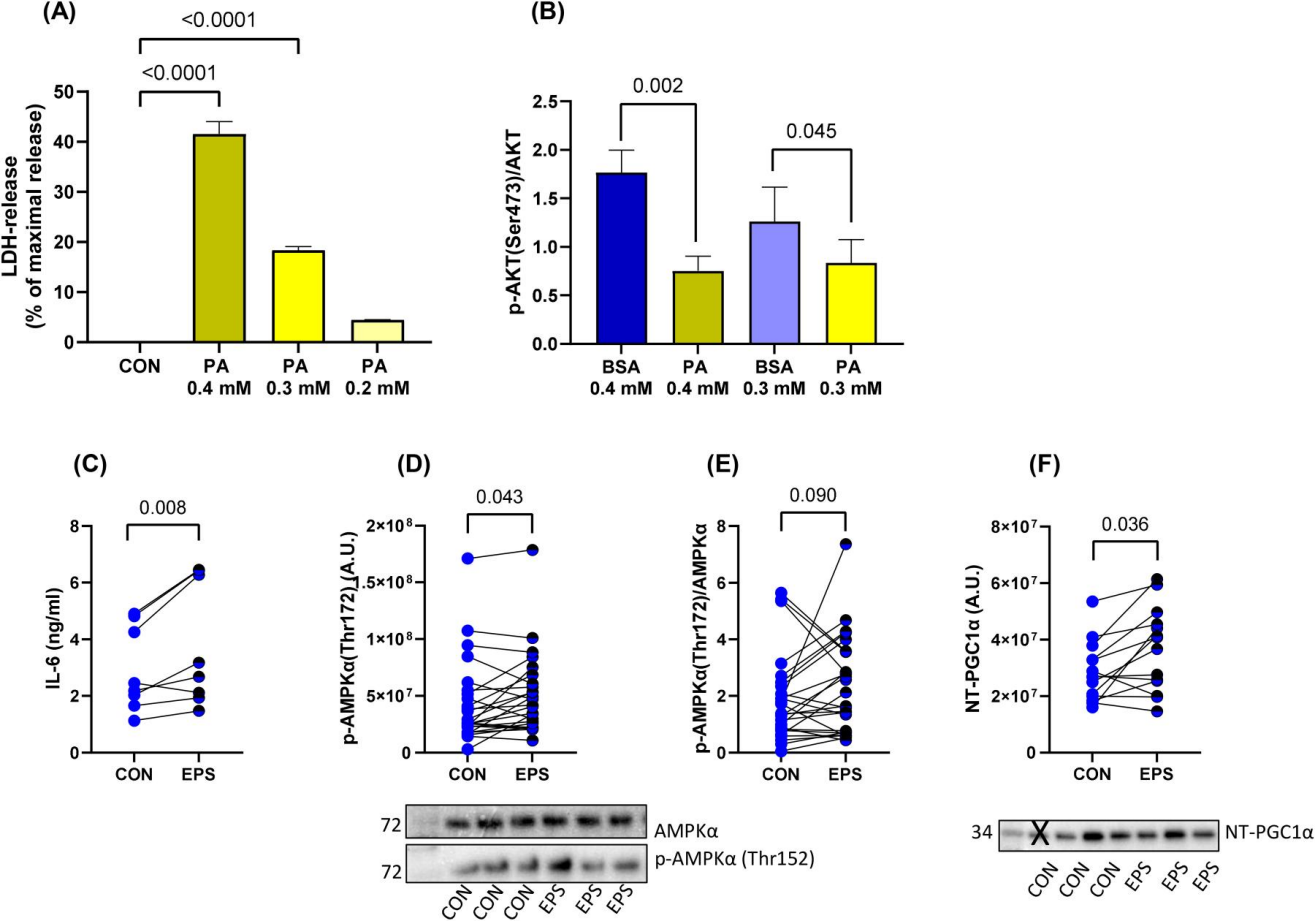
Electrical pulse stimulation induces an exercise‐like response in differentiated myotubes. (A) LDH concentration in the cell culture medium of cells treated with different concentrations of PA (*n* = 3); (B) Western blot analysis of p‐AKT(Ser473)/AKT in cells treated with different concentrations of PA (*n* = 3); (C) IL‐6 secretion in the cell culture medium of CON cells before and after EPS (*n* = 8); (D) Western blot analysis and representative images of p‐AMPKα(Thr172) (*n* = 26), (E) p‐AMPKα(Thr172)/AMPKα (*n* = 25–26) and (F) NT‐PGC (*n* = 14) protein expression in CON and EPS‐treated cells; *p*‐value is based on paired *t*‐test.

### 3.2 Electrical Pulse Stimulation Induces an Exercise‐Like Response in Differentiated Myotubes

Next, we confirmed that EPS did not affect cell viability by measuring LDH release (Figure S1D). To verify whether our EPS protocol mimics the characteristics of in vivo exercise, IL‐6 release in the cell culture medium and activation of AMP‐activated protein kinase α (AMPKα) were measured. As expected, EPS upregulated IL‐6 secretion, which reached a concentration of 3.8 ± 2.2 ng/ml compared with 2.9 ± 1.5 ng/ml in CON (Figure 1C). AMPKα(Thr172) phosphorylation increased upon EPS, and the p‐AMPKα(Thr172)/AMPKα ratio showed a trend toward increase (*p* = 0.090; Figure 1D,E). Importantly, EPS resulted in an increase of 28% of N‐terminal PGC1α in CON cells (NT‐PGC1α; Figure 1F), indicating activation of exercise‐like metabolic signaling (Jäger et al. 2007; Wen 2014). We confirmed the increase of IL‐6 secretion following EPS also in PA‐treated cells (Figure S1E). To investigate whether EPS modulates metabolic signaling under insulin‐resistant conditions, we calculated the exercise‐induced changes in AMPKα activation and NT‐PGC1α within each group (CON and PA) and compared the differences (Δ‐values) between the presence (EPS) and absence of EPS (baseline, BSL). The changes in the p‐AMPKα(Thr172)/AMPKα ratio between EPS and BSL were numerically lower in PA than in CON cells, although this difference did not reach statistical significance (CON, 0.40 ± 1.6; PA, −0.07 ± 0.93; Figure S1F), indicating a blunted response in PA‐treated cells. Notably, EPS did not induce an increase of NT‐PGC1α in PA cells, as reflected by a negative Δ‐value that was lower in PA cells compared to CON (Figure S1G).

### 3.3 Electrical Pulse Stimulation Partially Restores Proximal Insulin Signaling in Palmitate‐Treated Myotubes

To assess the effects of EPS on PA‐induced insulin resistance, insulin‐stimulated phosphorylation of AKT at Ser473 and Thr308 was analyzed in C2C12 myotubes. PA treatment did not change total AKT, but markedly reduced p‐AKT at both Ser473 and Thr308 (Figure 2A–C) and the ratio of p‐AKT(Ser473)/AKT as well as p‐AKT(Thr308)/AKT by 51% and 54%, respectively, compared with CON (Figure 2D,E). EPS partially rescued the PA‐induced insulin resistance, with p‐AKT(Ser473) and p‐AKT(Thr308) increased by 21% and 36% compared with PA, indicating that EPS attenuates the PA‐induced inhibition of AKT (Figure 2D,E). However, EPS did not affect AKT phosphorylation in CON cells. EPS also increased total IRS1 and p‐IRS1(Ser1101) (Figure 2F,G) in both CON and PA cells; however, the ratio of p‐IRS1(Ser1101)/IRS1 was unchanged following PA or EPS (Figure 2H).

**Figure 2:**
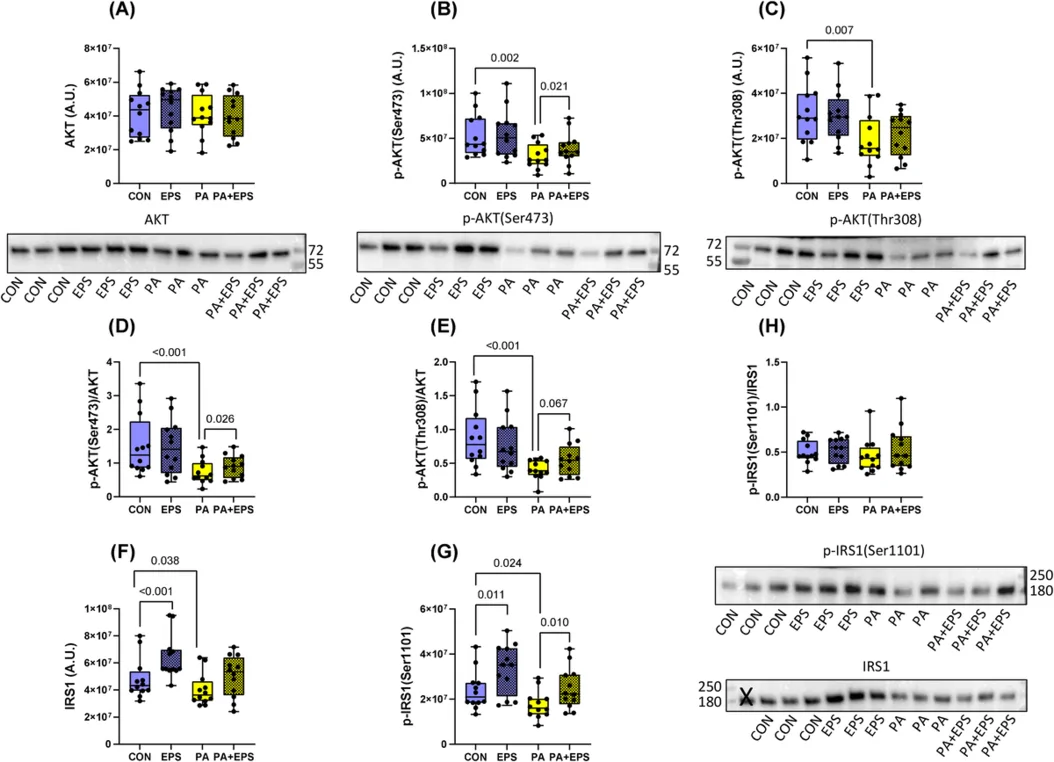
Electrical pulse stimulation partially attenuates insulin resistance but it does not reduce accumulation of lipotoxic intermediates in PA‐cells. Western blot analysis and representative images of (A) AKT (*n* = 12), (B) p‐AKT(Ser473) (*n* = 12), (C) p‐AKT(Thr308) (*n* = 12), (D) p‐AKT(Ser473)/AKT (*n* = 12), (E) p‐AKT(Thr308)/AKT (*n* = 12), (F) IRS1 (*n* = 12), (G) p‐IRS1(Ser1101) (*n* = 12) and (H) pIRS1(Ser1101)/IRS1 (*n* = 12) in CON, EPS‐, PA‐ and EPS + PA‐treated cells; *p*‐value is based on post‐hoc pairwise *t*‐test or paired Wilcoxon signed‐rank test with Bonferroni multiple comparison adjustment.

To further investigate signaling pathways associated with the beneficial effects of EPS under lipotoxic conditions, we assessed phosphorylation of acetyl‐CoA carboxylase (ACC), a downstream target of AMPKα involved in the regulation of fatty acid metabolism. Neither EPS nor PA affected the levels of ACC (Figure S2A). PA decreased the levels of p‐ACC(Ser69) by 26%, whereas EPS tended to increase p‐ACC(Ser69) and the p‐ACC(Ser69)/ACC ratio in PA‐treated myotubes compared with PA alone (PA + EPS vs PA, *p* = 0.072 and *p* = 0.084, respectively; Figure S2B,C). Taken together, these findings suggest that EPS promotes AMPKα‐related metabolic signaling under insulin‐resistant conditions, potentially contributing to the partial restoration of metabolic function in PA‐treated myotubes. Finally, we measured AS160 and phosphorylation of AS160, as downstream readouts of insulin signaling (Cartee 2015), which revealed that AS160 tended to decrease in PA‐treated cells (PA vs CON, *p* = 0.063) and that the p‐AS160(Thr642)/AS160 ratio increased upon EPS in CON cells (Figure S2D–F).

### 3.4 Electrical Pulse Stimulation Does Not Reduce Palmitate‐Induced Accumulation of Lipotoxic Intermediates

To further explore whether EPS ameliorates insulin resistance via reduction of IMCL, total DAG and CER content as well as their species, were quantified in C2C12 cells upon PA and EPS.

The total CER content increased following PA compared with CON, but EPS was not able to alter CER content, neither in CON nor in PA‐treated cells (Figure 3A). Similarly, all CER species were upregulated in PA‐treated cells in comparison to CON, but not influenced by EPS in both conditions (Figure S3).

**Figure 3:**
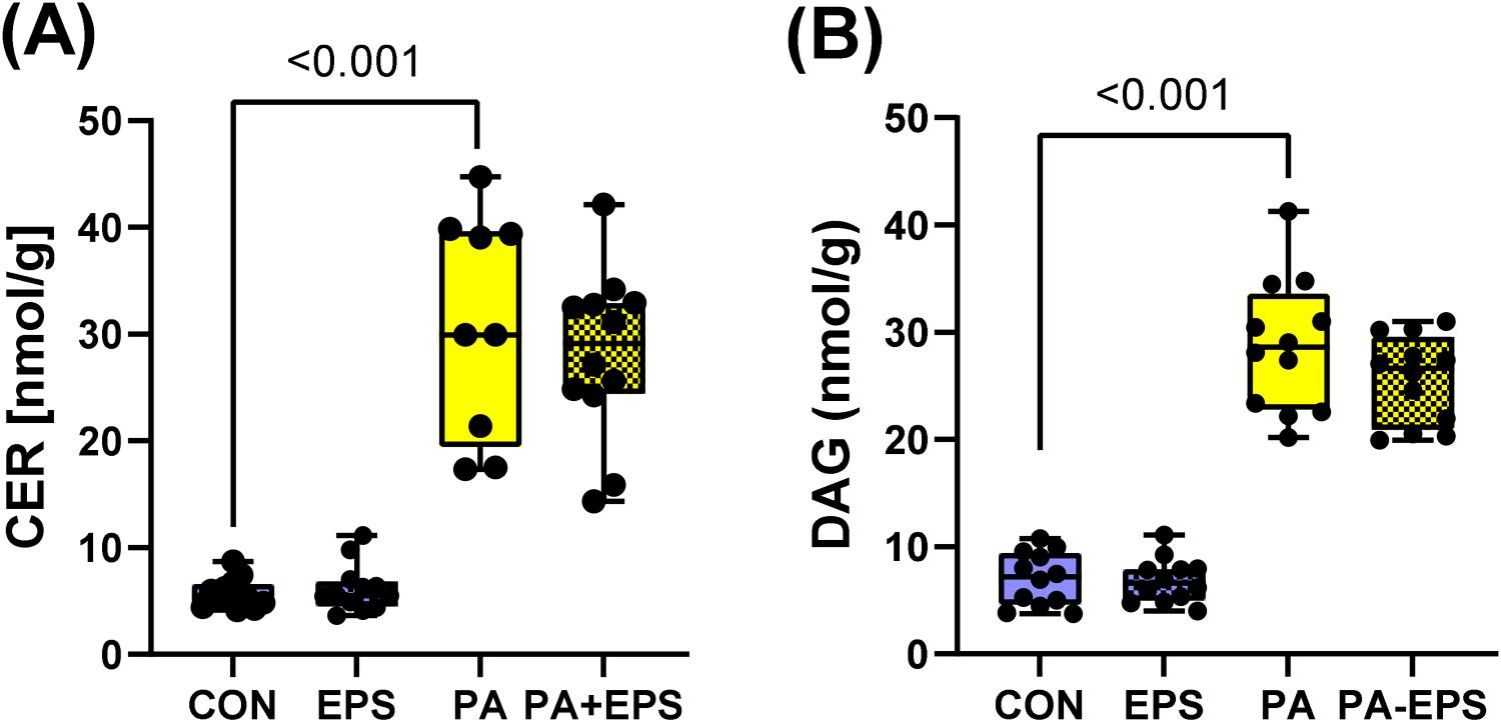
Palmitate‐induced accumulation of lipotoxic intermediates is not affected by EPS. Content of (A) total CER (*n* = 12) and (B) total DAGs (*n* = 12) in CON, EPS‐, PA‐ and EPS + PA‐treated cells; *p*‐value is based on post‐hoc pairwise *t*‐test or paired Wilcoxon signed‐rank test with Bonferroni multiple comparison adjustment.

A similar pattern was observed for DAG accumulation. Total DAG content was significantly increased in PA‐treated cells compared with CON, with no significant difference upon EPS (Figure 3B). Analysis of individual DAG species revealed significant increases of all DAG species in PA‐treated cells and no changes upon EPS, except of DAG(18:1/18:1), which was reduced in insulin‐resistant cells (Figure S4).

### 3.5 Electrical Pulse Stimulation Does Not Alter Mitochondrial Respiration but Affects Proteins Involved in Mitochondrial Dynamics

Next, we examined the effects of EPS on mitochondrial oxidative capacity in CON and in PA‐treated cells. Mitochondrial respiration (corrected for cell number) was not affected by PA treatment compared to CON. Additionally, EPS did not affect mitochondrial respiration on any substrate, neither in CON nor in PA‐treated cells (Figure S5).

As exercise could modulate insulin resistance via mitochondrial alterations (Koliaki and Roden 2016), we assessed the content of regulatory proteins of mitochondrial dynamics in CON as well as upon PA and EPS treatment. PA did not induce any change in any of the markers compared to CON cells, except for a reduction in mitochondrial fission 1 (FIS1; Figure 4A). However, the protein content of the mitochondrial fusion markers MFN1 and MFN2 were reduced by 20% and 15% respectively, upon EPS in PA (Figure 4B,C), whereas OPA1 was not affected by EPS neither in CON nor in PA‐treated cells (Figure S6A). As OPA1 has a long and short isoform, with the former involved in fusion, we also determined those isoforms. Interestingly, the long isoform of OPA1 showed a trend toward decrease upon EPS in PA‐treated cells (PA vs PA + EPS, *p* = 0.065; Figure 4D), whereas the short isoform was unchanged among groups (data not shown).

**Figure 4:**
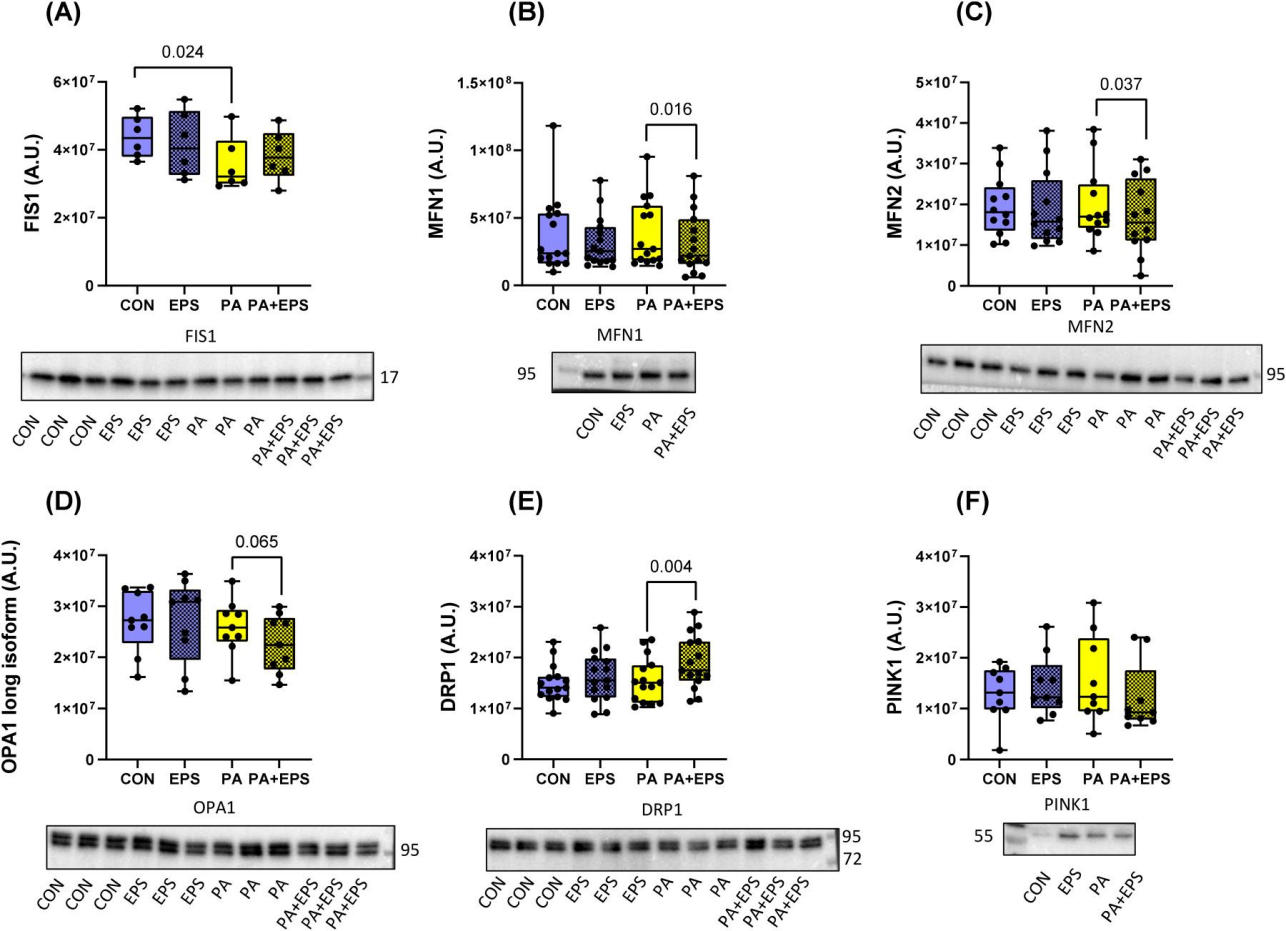
Expression of markers of mitochondrial fusion are reduced in PA cells upon EPS. Western Blot analysis and representative images of (A) FIS1 (*n* = 12), (B) MFN1 (*n* = 12), (C) MFN2 (*n* = 12), (D) OPA1 long isoform (*n* = 9), (E) DRP1 (*n* = 15) and (F) PINK1 (*n* = 9) in CON, EPS‐, PA‐ and EPS + PA‐treated cells; p‐value is based on post‐hoc pairwise *t*‐test or paired Wilcoxon signed‐rank test with Bonferroni multiple comparison adjustment.

Furthermore, EPS did not affect the the mitochondrial fission factor (MFF; *p* = 0.0614), which recruits DRP1 to initiate fission (Liu and Chan 2015) (Figure S6B), but significantly increased the content of DRP1 in PA‐treated cells (Figure 4E), although DRP1(Ser616) phosphorylation and the ratio of p‐DRP1(Ser616) to DRP1 were unchanged across groups (Figure S6C,D). Finally, the abundance of the mitophagy protein PINK1 was affected neither by PA nor by EPS (Figure 4F).

### 3.6 Eps Does Not Attenuate Endoplasmic Reticulum (ER) Stress in Lipid‐Induced Insulin Resistance Conditions

Since mitochondrial function or lipid species could not explain the effect of EPS on lipid‐induced insulin resistant, we next determined markers of ER stress, which represents an additional mechanism contributing to insulin resistance (Koh et al. 2013). Thus, we measured markers of the unfolded protein response (UPR). We found that the ER‐resident chaperone BiP (Binding immunoglobulin protein) was higher in PA‐treated cells compared with CON cells and tended to increase even further in cells treated with PA + EPS (*p* = 0.054; Figure 5A). Conversely, inositol requiring enzyme 1 (IRE1α) levels, the ratio of phosphorylated eukaryotic initiation factor‐2α (p‐eIF2α(Ser51)) to total eIF2α and C/EBP homologous protein (CHOP) levels remained unchanged across CON, EPS, PA, and EPS + PA‐treated cells (Figure 5B–D).

**Figure 5:**
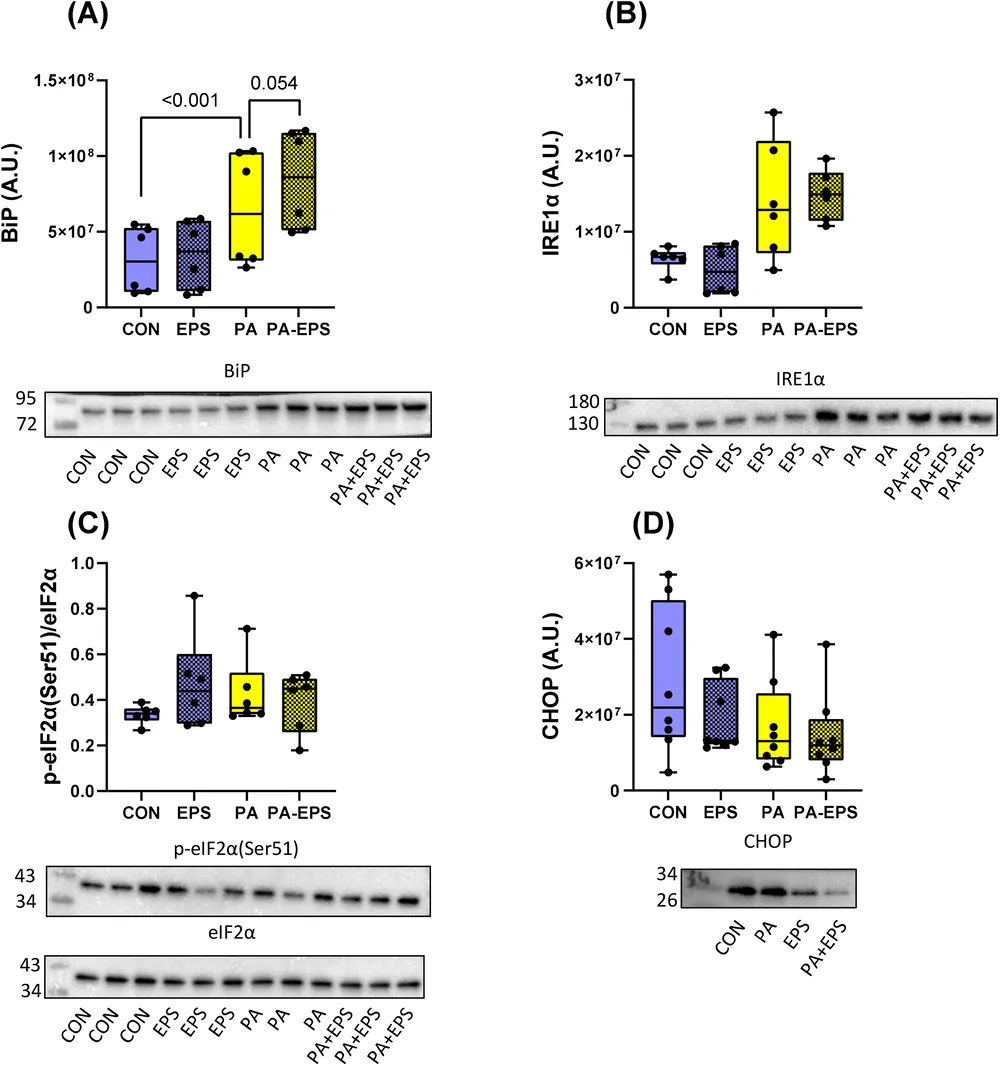
Electrical pulse stimulation does not reduce ER stress. Western Blot analysis and representative images of (A) BiP (*n* = 6), (B) IRE1α (*n* = 6), (C) peIF1α/eIF1α (*n* = 6) and (D) CHOP (*n* = 8) in CON, EPS‐, PA‐ and EPS + PA‐treated cells; *p*‐value is based on post‐hoc pairwise *t*‐test or paired Wilcoxon signed‐rank test with Bonferroni multiple comparisons adjustment.

### 3.7 Eps‐Stimulated Cells Secrete Factors Which Protect From Impaired AKT Phosphorylation Under Conditions of Lipid‐Induced Insulin Resistance

Since intracellular changes did not fully account for the beneficial effects of EPS on PA‐induced insulin resistance, we investigated whether autocrine signaling via the myotube secretome contributes to these improvements. Conditioned media (CM) were collected from CON cells stimulated for 24 h in the presence or absence of EPS (CM‐CON and CM‐EPS) and used to treat CON and PA‐treated cells. CM from both CON and EPS cells reduced the content of total AKT in PA‐treated cells but did not affect the content of p‐AKT(Ser473) and p‐AKT(Thr308) among conditions (Figure S7A–C). We confirmed that PA induced a 40% reduction in insulin‐stimulated p‐AKT(Ser473)/AKT ratio compared with CON cells (Figure 6A). Interestingly, incubation of PA‐treated cells with CM‐EPS reduced AKT(Ser473) phosphorylation by only 5% compared with CON, indicating that CM‐EPS attenuated the PA‐induced impairment of AKT activation. CM‐CON did not affect PA‐induced insulin‐stimulated AKT activation. Notably, AKT(Ser473) phosphorylation remained unaltered in CON cells incubated with CM‐EPS (Figure 6A). Phosphorylation of AKT(Thr308) did not change among the experimental conditions (Figure 6B).

**Figure 6:**
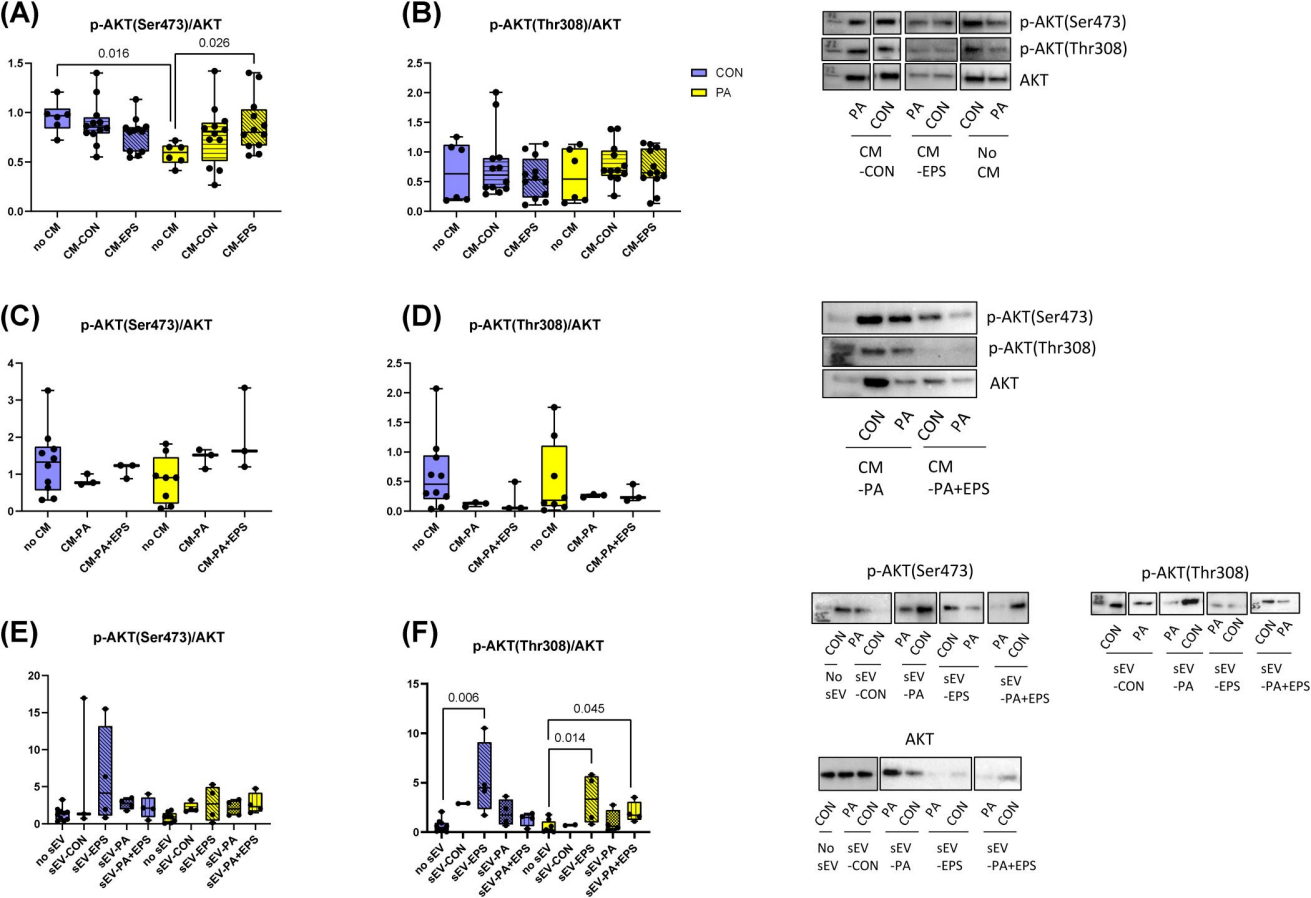
EPS‐stimulated cells secrete factors which protect from impaired AKT phosphorylation under conditions of lipid‐induced insulin resistance. Western blot analysis and representative images of (A) p‐AKT(Ser473)/AKT (*n* = 12; *n* = 6 for CON and PA no CM) and (B) p‐AKT(Thr308)/AKT (*n* = 12; *n* = 6 for CON and PA no CM) in CON and PA‐cells receiving CM from CON or EPS cells; (C) p‐AKT(Ser473)/AKT (*n* = 3; *n* = 10 for CON and PA no CM) and (D) p‐AKT(Thr308)/AKT (*n* = 3; *n* = 10 for CON and PA no CM) in CON and PA‐cells receiving CM from PA or PA + EPS treated cells; (E) p‐AKT(Ser473)/AKT (*n* = 4; *n* = 10 for CON and PA no sEV) and (F) p‐AKT(Thr308)/AKT (*n* = 4; *n* = 10 for CON and PA no sEV) in CON and PA‐cells receiving sEV from CON, EPS, PA and PA + EPS treated cells; *p*‐value is based on post‐hoc pairwise *t*‐test or Dunn test with Bonferroni multiple‐comparison adjustment.

Next, we incubated CON and PA‐treated cells with CM collected from PA‐treated cells stimulated for 24 h in the presence or absence of EPS (CM‐PA and CM‐PA + EPS). Our preliminary data show that the content of total AKT, p‐AKT(Ser473) and p‐AKT(Thr308) did not differ across conditions (Figure S7D–F). However, CM from PA + EPS cells induced a numerical increase of p‐AKT(Ser473)/AKT ratio in PA recipient cells compared to PA cells without CM and PA cells receiving CM‐PA (A.U. ±SD: PA no CM, 1.32 ± 0.89; PA + CM‐PA, 1.44 ± 0.27; PA + CM‐PA + EPS, 2.05 ± 1.13; Figure 6C). The p‐AKT(Thr308)/AKT was reduced in both CON and PA‐treated cells receiving CM‐PA and CM from PA + EPS cells (Figure 6D).

To further investigate which factor contributes to the protective effects of the EPS induced secretome, sEV were isolated from conditioned media of CON (sEV‐CON), EPS (sEV‐EPS), PA (sEV‐PA), and PA + EPS (sEV‐PA + EPS) myotubes and transferred to CON and PA recipient cells. sEV from EPS‐stimulated cells decreased AKT in CON and PA‐treated cells, whereas the content of p‐AKT(Ser473) was not affected by any sEV. In contrast, p‐AKT(Thr308) was numerically higher in cells receiving sEV‐EPS than in untreated cells under both CON and PA conditions (A.U. ±SD: CON no sEV, 7.05 ± 0.38; CON + sEV‐EPS, 7.52 ± 0.17; PA, 6.86 ± 0.47; PA + sEV‐EPS, 7.18 ± 0.13), although this difference did not reach statistical significance (Figure S8A–C). Of note, sEV did not alter the p‐AKT(Ser473)/AKT ratio (Figure 6E), whereas sEV‐EPS and sEV‐PA + EPS increased p‐AKT(Thr308)/AKT ratio in both CON and PA‐treated cells (Figure 6F).

## 4 Discussion

Overall, we show that EPS partially rescues PA‐induced impairment of AKT signaling while altering the abundance of proteins involved in mitochondrial fusion and fission, suggesting changes in the regulation of mitochondrial dynamics under lipotoxic stress.

Moreover, parallel assessment of multiple intracellular and extracellular pathways revealed that EPS improves AKT signaling through mechanisms independent of changes in intracellular lipid accumulation or mitochondrial respiratory function, implicating exercise‐induced secreted factors, including sEV, as potential mediators.

In humans, acute lipid infusion increases intramyocellular DAG levels, which is associated with activation of novel protein kinase C (nPKC) θ, increased inhibitory IRS1 serine phosphorylation, and reduced insulin‐stimulated AKT phosphorylation (Szendroedi et al. 2014). Consistent with this, consumption of PA‐rich diet can acutely induce myocellular insulin resistance, which is associated with activation of nPKCs and inhibition of insulin signaling (Sarabhai et al. 2022). In our study, PA treatment for 24 h induced an impairment of proximal insulin signaling, as evidenced by reduced AKT phosphorylation, and accumulation of the bioactive lipid species DAG and CER. Interestingly, some of the DAG species increased in our study, such as DAG(18:0/16:0) and DAG(18:0/20:4), have previously been associated with activation of nPKCε, particularly when enriched in the membrane fraction of SkM, and insulin resistance (Bergman et al. 2012). However, we did not observe increased ratio of p‐IRS1(Ser1101)/IRS1despite the accumulation of these DAGs species. This discrepancy may reflect, at least in part, differences in subcellular lipid partitioning rather than total lipid abundance. As the signaling effects of DAG and CER are highly dependent on their distribution on cellular compartments, potential compartment‐specific changes may have remained masked. Interestingly, CER, particularly C16:0 and C18:0, have been shown to impair insulin signaling through mechanisms downstream of IRS1, including inhibition of AKT activation via protein phosphatase 2A (PP2A) or nPKCζ (Sarabhai et al. 2022; Stratford et al. 2001; Turpin et al. 2014; Stratford et al. 2004; Powell et al. 2003). Thus, the reduced AKT phosphorylation observed in our study may be more consistent with CER‐mediated inhibition of insulin signaling, although future studies would need to perform compartment‐specific lipid analysis to determine the relative contribution of distinct DAG and CER species.

Lipid‐induced insulin resistance has also been linked to alterations in mitochondrial function, although the available evidence remains inconsistent. In humans, increased lipid availability during lipid infusion has been shown to impair insulin‐stimulated ATP synthesis in SkM, suggesting that elevated fatty acid levels may negatively affect mitochondrial oxidative capacity (Brehm et al. 2006). However, other studies reported that acute elevation of plasma lipids reduces glucose disposal without affecting mitochondrial ATP synthesis, indicating that lipid‐induced insulin resistance may occur independently of direct impairments of mitochondrial oxidative phosphorylation capacity (Brehm et al. 2010). In contrast, endurance‐trained individuals with high mitochondrial oxidative capacity are partially protected against lipid‐induced insulin resistance despite elevated IMCL, suggesting that exercise‐induced mitochondrial adaptations may attenuate the metabolic consequences of lipid overload (Phielix et al. 2012).

Exercise is well known to improve SkM insulin sensitivity and glucose metabolism. These adaptations are mediated by multiple mechanisms, including repartitioning of IMCL, enhancement of mitochondrial biogenesis and remodelling of the mitochondrial network via fusion and fission (Mastrototaro et al. 2024; de Almeida et al. 2023). In this context, EPS has emerged as a useful tool to model contractile activity in vitro and to examine exercise‐induced signaling events and molecular adaptations in SkM (Nikolić et al. 2017). EPS increased the secretion of the myokine IL‐6 in control and PA‐induced C2C12 myotubes, indicating that secretory response to EPS is preserved under lipotoxic conditions (Lambernd et al. 2012; Pedersen and Fischer 2007; Förster et al. 2025). Furthermore, in control cells, EPS increased AMPKα activation and NT‐PGC1α abundance, consistent with activation of an exercise‐like metabolic signaling (Gibala et al. 2009; Spaulding and Yan 2022). In contrast, insulin‐resistant cells showed unchanged NT‐PGC1α levels and attenuated AMPKα response to EPS, despite increased p‐ACC(Ser69) levels. These findings suggest that lipotoxic stress selectively impairs intracellular contraction‐induced metabolic adaptations, consistent with previous findings in myotubes derived from obese individuals (Park et al. 2019). Nevertheless, EPS partially rescued AKT activation in lipid‐induced insulin‐resistant cells, indicating that improvements in proximal insulin signaling can occur despite limited metabolic remodeling. This apparent dissociation suggest that PA‐treated cells retain selected EPS‐responsive pathways relevant for insulin signaling, whereas broader mitochondrial respiratory and AMPKα/PGC1α‐related adaptations remain impaired. In this context, the rescue of AKT phosphorylation may involve mechanisms that are at least partly independent of NT‐PGC1α induction, including alterations in proteins involved in mitochondrial dynamics and autocrine effects of contraction‐induced factors. This interpretation is supported by our CM and sEV experiments and is consistent with in vivo evidence showing that improvements in whole‐body and hepatic insulin resistance following 12‐week high intensity interval training (HIIT) occur predominantly in insulin‐resistant individuals, including those with or without T2DM, but not in insulin‐sensitive persons (Apostolopoulou et al. 2021).

In contrast to in vivo exercise, which improves SkM mitochondrial oxidative capacity (Trinks 2026; Batterson et al. 2023), EPS did not enhance mitochondrial respiration neither in control nor in PA myotubes, despite increased mitochondrial biogenesis in control cells. Of note, the lack of improved mitochondrial respiration in PA cells was consistent with the unchanged levels of NT‐PGC1α. Nevertheless, EPS affected proteins involved in mitochondrial dynamics under PA conditions. While lipid overload has been associated with mitochondrial fragmentation and a pro‐fission phenotype in SkM cells (Jheng et al. 2012), exercise‐induced regulation of mitochondrial dynamics appears to be more complex than a simple shift toward fusion. Acute endurance exercise has been shown to increase the Mfn1 and Mfn2 transcripts (Cartoni et al. 2005), as well as DRP1‐Ser616 phosphorylation in human SkM (Moore et al. 2019; Kruse et al. 2017; Schwalm et al. 2017; Ritenis et al. 2025), suggesting that exercise promotes dynamic mitochondrial remodeling rather than exclusively favouring fusion. Under lipotoxic conditions, we observed increased DRP1 protein abundance following EPS, whereas DRP1‐Ser616 phosphorylation remained unchanged. In parallel, EPS reduced MFN1 and MFN2 in PA‐treated cells, consistent with previous findings in EPS‐stimulated primary myotubes from obese individuals, where fusion markers were also reduced (Kugler et al. 2021). Together, these findings support the view that contractile activity under insulin resistant or lipotoxic conditions may induce a coordinated adjustment of mitochondrial dynamics proteins rather than a uniform response favouring fusion.

Previous studies have shown that exercise may decrease DAG and CER levels (Shepherd et al. 2017), although this has not been consistently observed across studies (Mendham et al. 2021; Mezincescu et al. 2024). We recently showed that DAG levels increased in endurance‐trained athletes compared with non‐trained controls (Pesta et al. 2025) and that 12‐week HIIT does not alter the total CER content but leads to elevated CER species in insulin‐resistant, but not insulin‐sensitive individuals (Hendlinger et al. 2025). In the present study, DAG and CER accumulation was not affected by EPS, neither under control nor insulin‐resistant conditions. Thus, the partial restoration of AKT signaling following EPS does not appear to result from a reduction in total cellular lipotoxic lipid accumulation. However, because the signaling effects of DAG and CER depend not only on their total abundance, but also on their subcellular distribution between membrane‐associated pools, lipid droplets and other intracellular compartments (Roden and Shulman 2019; Mastrototaro and Roden 2021), we cannot exclude the possibility that EPS altered specific lipid pools or compartmental distribution despite unchanged total cellular DAG and CER content.

Since our results did not point towards direct effects of EPS on PA‐induced changes in lipid levels, mitochondrial respiratory capacity or ER stress, we investigated if autocrine effects may be involved in the blunting of PA‐induced insulin resistance by EPS. Thus, it is known that both aerobic and resistance training trigger the release of several myokines, such as irisin, apelin and myostatin, as well as sEV which can mediate organ crosstalk and improve lipid metabolism, fatty oxidation, inflammatory pathways and systemic insulin sensitivity (Mastrototaro and Roden 2024; Chow et al. 2022). Here, we indeed show that CM from EPS‐treated cells attenuated the PA‐induced impairment of AKT activation, with Ser473 phosphorylation reaching levels comparable to the CON cells. CM from PA + EPS treated myotubes also showed a tendency to improve AKT activation in PA recipient cells, suggesting that EPS may partially preserve the secretory response under lipotoxic conditions. Furthermore, the transfer of sEV from EPS‐stimulated myotubes reproduced aspects of this protective effect, suggesting that sEV‐associated signals may contribute to the beneficial actions of the EPS‐induced secretome. Interestingly, sEV treatment was associated with AKT Thr308 phosphorylation, suggesting that soluble factors and sEV cargo may engage distinct regulatory mechanisms within the AKT signaling pathway. However, these findings require further validation in studies with larger sample sizes to confirm the contribution of EPS‐induced secreted factors and sEV‐associated signals to the observed protective effects. Together, these findings identify a potential autocrine pathway through which exercise benefits SkM independently of mitochondrial respiration, lipid handling or ER stress.

A strength of the present study is the integrated assessment of insulin signaling together with lipidomic profiling, mitochondrial respiration, proteins involved in mitochondrial dynamics, ER stress markers as well as CM and sEV experiments within the same lipotoxic EPS model, which allowed to evaluate multiple intracellular and extracellular pathways in parallel. Some limitations of this study need to be considered. First, insulin signaling was only assessed under insulin‐stimulated conditions and our assessment was limited to proximal insulin signaling. Therefore, further studies are required to determine whether EPS also restores downstream insulin signaling and fully rescues insulin sensitivity. Second, mitochondrial dynamics were assessed based on the protein abundance of key regulatory markers involved in mitochondrial fusion, fission and mitophagy, without direct visualization of mitochondrial network architecture due to constraints inherent to the EPS induction system. Therefore, our conclusions should be interpreted as alterations in the regulation of mitochondrial dynamics rather than evidence of structural remodelling. In addition, in the absence of a validated mouse‐specific antibody for p‐PINK1(Thr257), PINK1 was assessed as a marker of mitophagy. As the currently available PARKIN antibody did not yield reliable signals, conclusions regarding mitophagy should be interpreted with caution (Mastrototaro et al. 2024; Drake et al. 2019). Third, only total cellular DAG and CER content was measured, without subcellular distribution. Given that the biological effects of lipid species depend on their intracellular localization, changes in specific lipotoxic pools or membrane‐associated fractions may have remained undetected. Fourth, not all ER stress markers were measured because of the limited availability of mouse‐specific antibodies. Fifth, the contribution of secreted factors and sEV released from EPS‐ and PA + EPS‐treated myotubes remains an exploratory finding and requires further validation in larger sample sizes and assessment of additional components of insulin signaling pathway as well as functional measures of insulin action, to determine whether the effects of EPS‐induced secreted factors and sEVs extend beyond AKT signaling. Similarly, the cargo composition of sEV needs further characterization. Finally, the study was performed in immortalized murine C2C12 myotubes, which represent a well‐established and reproducible *in vitro* model to investigate contraction‐induced signaling, lipid‐induced insulin resistance and myocellular adaptations. However, C2C12 myotubes do not fully recapitulate the phenotype, metabolic flexibility and donor‐specific characteristics of primary human skeletal muscle cells; therefore, future studies in primary human skeletal muscle cells would be valuable to determine the extent to which these observations translate to human physiology.

Overall, our findings demonstrate that EPS partially restores PA‐induced impairment of AKT activation in C2C12 myotubes. This effect is accompanied by altered mitochondrial dynamics, characterized by reduced fusion proteins and increased DRP1 levels, suggesting remodelling of the mitochondrial network. In addition, conditioned medium and sEV from EPS‐stimulated cells modulate AKT phosphorylation, indicating that contraction‐induced released factors may contribute to the restoration of insulin‐responsive AKT signaling.

In conclusion, these results support the concept that secretome‐mediated crosstalk and changes in proteins regulating mitochondrial dynamics may act together to ameliorate SkM insulin sensitivity under lipotoxic conditions.

## Author Contributions

**Falcone Alessandro:** investigation, writing – original draft, writing – review and editing. **Yilmaz Sevgican:** investigation, writing – review and editing, formal analysis, visualization. **Hendlinger Mona:** investigation, writing – review and editing. **Schrauwen Patrick:** writing – review and editing. **Lipaeva Polina:** visualization, formal analysis, writing – review and editing. **Piribauer Marlene:** investigation, writing – review and editing. **Heilmann Geronimo:** investigation, writing – review and editing. **Trenkamp Sandra:** investigation, writing – review and editing. **Roden Michael:** writing – original draft, writing – review and editing, supervision, resources, funding acquisition, methodology, conceptualization. **Mastrototaro Lucia:** conceptualization, writing – original draft, writing – review and editing, investigation, supervision, methodology, resources, visualization, funding acquisition.

## Conflicts of Interest

MR received lecture fees or served on advisory boards for Astra Zeneca, Boehringer‐Ingelheim Pharma, Eli Lilly, Madrigal, MSD, Novartis, Novo Nordisk, Sanofi US and Target RWE, and performed investigator‐initiated research with support from Boehringer‐Ingelheim Pharma and Novo Nordisk to the German Diabetes Center (DDZ).

PS received speaking fees or served on advisory boards for AstraZeneca, Medimmune, Rivus Pharmaceuticals, and Unilever, received support for investigator‐initiated research from AstraZeneca, Medimmune, Pfizer, and Unilever, and is a member of the editorial board of Diabetologia. All other authors declare no competing interests.

## Supporting information


Supporting File 1

Supporting File 2

## Data Availability

All data generated or analysed during this study are included in this published article and its supplementary information files.
