# Supplementary material for: Electrical Pulse Stimulation Partially Restores Insulin Signaling in Palmitate‐Induced Insulin‐Resistant Skeletal Muscle Cells via Mitochondrial Remodeling and Contractile Activity‐Dependent Secretome
Source: J Cell Physiol. 2026 Oct 6;241(10):e70240. doi: 10.1002/jcp.70240 (PMC13640965; doi:10.1002/jcp.70240)
Supplement: Supplementary file 1 [file JCP-241-0-s001.docx]

**Fig. S1. (A)** Differentiation protocol for C2C12 cells; **(B)** Western Blot analysis of myosin heavy chain 3 in C2C12 myotubes initially seeded at different densities; **(C)** Bright field imaging of undifferentiated mononucleated myoblasts and multinucleated myotubes; **(D)** LDH concentration in the cell culture medium of cells stimulated with EPS (n=3); **(E)** IL-6 release in PA cells before and after EPS (n=8); **(F)** Changes in AMPKα activation (n=25 in CON and n=24 in PA) and **(G)** NT-PGC1α (n=14) both expressed as the Δ between values after EPS and before EPS (EPS–BSL), in CON and PA cells; p-values are based on paired t-test.

**Fig. S2.** Western blot analysis and representative images of ACC (n=8) **(A)**, p-ACC(Ser69) (n=8) **(B)**, p-ACC(Ser69)/ACC (n=8) **(C)**, AS160 (n=8) **(D)**, p-AS160(Thr642) (n=5-7) **(E)** and p-AS160(Thr642)/AS160 (n=5-7) **(F)** in CON, EPS, PA and PA+EPS cells (n=8); p-values are based on post-hoc pairwise t-test or paired Wilcoxon signed-rank test with Bonferroni multiple-comparison adjustment.

**Fig. S3.** CER species in CON, EPS, PA and PA+EPS cells (n=9-12); p-values are based on post-hoc pairwise t-test or paired Wilcoxon signed-rank test with Bonferroni multiple-comparison adjustment.

**Fig. S4.** DAG species in CON, EPS, PA and PA+EPS cells (n=12); p-values are based on post-hoc pairwise t-test or paired Wilcoxon signed-rank test with Bonferroni multiple-comparison adjustment.

**Fig. S5.** Mitochondrial leak respiration with electron input through ETF **(A)**, ADP-stimulated respiration with electron input through ETF **(B)**, CI **(C)**, CI and CII **(D)**, leak respiration **(E)** and maximal uncoupled respiration **(F)** through ETF, CI and CII in CON, EPS, PA and PA+EPS cells (n=12); p-values are based on post-hoc pairwise t-test or paired Wilcoxon signed-rank test with Bonferroni multiple-comparison adjustment.

**Fig. S6.** Western blot analysis and representative images of OPA1 (n=9) **(A)**, MFF (n=12) **(B)**, p-DRP1(Ser616) (n=15) **(C)** and p-DRP1(Ser616)/DRP1 (n=15) **(D)** in CON, EPS, PA and PA+EPS cells; p-values are based post-hoc pairwise t-test or paired Wilcoxon signed-rank test with Bonferroni multiple-comparison adjustment.

**Fig. S7.** Western blot analysis of AKT, p-AKT(Ser473), and p-AKT(Thr308) in CON and PA cells treated with CM-CON and CM-EPS **(A-C)** (n=6 for CON and PA no CM, n=12 for CON and PA with CM) as well as with CM-PA and CM-PA+EPS (n=10 for CON no CM, n=8 for PA no CM, n=3 for CON and PA with CM) **(D-F)**; p-values are based on post-hoc pairwise t-test or Dunn test with Bonferroni multiple-comparison adjustment

**Fig. S8**. Western blot analysis of AKT **(A)**, p-AKT(Ser473) **(B)** and p-AKT(Thr308) **(C)** in CON and PA cells treated with sEV-CON, sEV-EPS, sEV-PA and sEV-PA+EPS (n=10 for CON no sEV, n=8 for PA no sEV, n=4 for CON and PA with sEV); p-values are based on post-hoc pairwise t-test or Dunn test with Bonferroni multiple-comparison adjustment.
